# Convenient Detection of the Citrus Greening (Huanglongbing) Bacterium ‘*Candidatus* Liberibacter asiaticus’ by Direct PCR from the Midrib Extract

**DOI:** 10.1371/journal.pone.0057011

**Published:** 2013-02-20

**Authors:** Takashi Fujikawa, Shin-Ichi Miyata, Toru Iwanami

**Affiliations:** National Institute of Fruit Tree Science, Tsukuba, Ibaraki, Japan; Nanjing Agricultural University, China

## Abstract

A phloem-limited bacterium, ‘*Candidatus* Liberibacter asiaticus’ (Las) is a major pathogen of citrus greening (huanglongbing), one of the most destructive citrus diseases worldwide. The rapid identification and culling of infected trees and budwoods in quarantine are the most important control measures. DNA amplification including conventional polymerase chain reaction (PCR) has commonly been used for rapid detection and identification. However, long and laborious procedures for DNA extraction have greatly reduced the applicability of this method. In this study, we found that the Las bacterial cells in the midribs of infected leaves were extracted rapidly and easily by pulverization and centrifugation with mini homogenization tubes. We also found that the Las bacterial cells in the midrib extract were suitable for highly sensitive direct PCR. The performance of direct PCR using this extraction method was not inferior to that of conventional PCR. Thus, the direct PCR method described herein is characterized by its simplicity, sensitivity, and robustness, and is applicable to quarantine testing.

## Introduction

Citrus greening (huanglongbing; HLB) is a devastating disease of citrus trees with high economical costs to the worldwide citrus industry. Symptoms include blotchy chlorosis and/or mottling of leaves; yellowish shoots; vein corking; stunted growth; poor root growth; small, green, and malformed fruits; and finally, death [1]. This disease is caused by phloem-limited fastidious bacteria, ‘*Candidatus* Liberibacter spp.’ [1], [2] and is transmitted by grafting and by the sap-sucking psyllids *Diaphorina citri* and *Trioza erytreae* [1]. There are three species identified as pathogens. They are ‘*Ca.* L. asiaticus’ (Las), ‘*Ca.* L. africanus,’ and ‘*Ca.* L. americanus’[1]–[5], and Las is in the most widespread (e.g., Asia, Brazil, and North America) [1], [6], [7]. All major commercial citrus cultivars are susceptible to this species, and no effective control is known other than the removal of infected trees. Therefore, in areas in which greening has not become established, rapid identification and culling of infected trees and budwoods in quarantine are the most important control measures.

Various DNA amplification methods, including conventional polymerase chain reaction (PCR), real-time PCR, nested PCR, and loop-mediated isothermal amplification, have been used to detect greening-infected plants [2], [8]–[17]. Conventional PCR is the preferred method because it is inexpensive and easy to perform. Improved conditions and Las-specific primers for conventional PCR are often reported and applied. In particular, conventional PCR using the Las-specific primer set Las606/LSS, which targets a specific part of Las 16S ribosomal DNA, is a highly sensitive and robust method for Las detection [18]. Conventional PCR requires that total DNAs from suspected tissues be extracted using DNA extraction kits or reagents before amplification. Therefore, when a large number of suspected trees are examined, the time and cost for DNA extraction make the process impractical. To overcome this problem, procedures for DNA extraction must be made as simple as possible.

Herein, we show that Las cells of citrus midribs were extracted and concentrated as part of a pellet by homogenization and centrifugation using mini homogenizer tubes. When direct PCR with the Las606/LSS primer set was performed using the resuspension of Las cells as templates, Las detection was successful much more efficiently than conventional PCR with the OI1/OI2c primer set that is commonly used in quarantine inspection. Thus, direct PCR using this Las extraction method would facilitate the identification of Las-infected trees within a large number of trees with suspected infection.

## Materials and Methods

### Las-infected citrus

Potted seedlings of rough lemon (*Citrus jambhiri* Lush.) were inoculated with Las by grafting and preserved in a quarantined greenhouse. The source of Las was flat lemon (*Citrus depressa* Hayata.) collected on the Ryukyu Islands in Okinawa Prefecture (Japan) as described by Furuya et al. [19] and Tomimura et al. [20]. Las-infected leaves (namely, Ishigaki1-A) and healthy leaves were used to prepare samples for FISH assay. For each PCR analysis of a sample, three leaves from individual rough lemon trees with or without symptoms were collected, and a total of 11 Las-infected samples (namely, Ishigaki1-A, Ishigaki1-B, Ishigaki1-C, Kin1-A, Kin1-B, Kin1-C, OK901-A, OK901-B, Ishigaki3-A, Ishigaki4-A, and Miyako13-A) and one control sample (healthy) were saved. The appearances of the leaves of each sample are shown in Fig. S1. Only five samples (Ishigaki1-B, Ishigaki1-C, Kin1-B, OK901-A and Ishigaki4-A) were symptomatic (see Fig. S1).

### Sample preparation for PCR

Each leaf sample was processed for PCR templates as described below. The midribs of three leaves in each sample were excised and chopped roughly. Approximately 0.05 g of midrib pieces were used for each of the four preparation methods.

#### Preparation 1

The midrib pieces were ground with a mortar and pestle before the total DNAs (citrus genomic DNA with Las genomic DNA) were extracted using a DNeasy plant mini kit (QIAGEN, Valencia, CA). This procedure was regarded as a typical DNA extraction techniques consisting of reagents and kits. The DNA solutions were prepared to a volume of 400 µL with distilled water. The sample prepared using this method was designated *Extracted DNA*.

#### Preparation 2

The midrib pieces were ground with a mortar and pestle and suspended in 400 µL distilled water. The sample prepared using this method was designated *Crude*.

#### Preparation 3

The midrib pieces were ground with a mortar and pestle and suspended in 400 µL distilled water. Next, they were shredded using a QIAshredder spin column (QIAGEN) with centrifugation for 2 min at 5000×*g*. After centrifugation, 400 µL flow- through solution was collected and designated *QIAshredder-flow-through*. Also, the precipitated pellet was resuspended in 400 µL distilled water and designated *QIAshredder-pellet*.

#### Preparation 4

The midrib pieces in 400 µL distilled water were homogenized using the mini homogenizer tube Biomasher III (Nippi, Tokyo, Japan) and centrifuged for 2 min at 5000×*g*. After centrifugation, 400 µL flow-through solution was collected and designated *Biomasher-flow-through*. Also, the precipitated pellet was resuspended in 400 µL distilled water and designated *Biomasher-pellet*.

### Conventional and direct PCR

Conventional and direct PCR were performed in a 20-µL reaction mixture with 1× PCR buffer containing 0.2 mM deoxyribonucleotide triphosphates (dNTPs), 0.25 µM each primer, 0.5 U *ExTaq* polymerase (Takara Bio Inc., Shiga, Japan), and 2 µl PCR templates. *Extracted DNA* sample obtained by described above *preparation 1* was adjusted to the concentration of 1 ng/µl and used as template for conventional PCR (2 ng amount DNA as template in PCR mixture), The other samples (*Crude*, *QIAshredder-flow-through*, *QIAshredder-pellet*, *Biomasher-flow-through*, and *Biomasher-pellet*) obtained described above *preparation 2*, *3*, and *4*, respectively, were used as templates for direct PCR. The PCR conditions were 9 min of predenaturation at 96°C, followed by 35 or 40 cycles of 30 s of denaturation at 96°C, 1 min of annealing at 55°C, 30 s of extension at 72°C, and a single final extension of 7 min at 72°C. The presence and amount of PCR products were confirmed with 1.5% agarose gel electrophoresis. Two sets of PCR primers targeting Las 16S ribosomal DNA were used in this study: one primer set was Las606/LSS (forward primer Las606 [5′-GGA GAG GTG AGT GGA ATT CCG A-3′] and reverse primer LSS [5′-ACC CAA CAT CTA GGT AAA AAC C-3′]) [18], and the other primer set was OI1/OI2c (forward primer OI1 [5′-GCG CGT ATG CAA TAC GAG CGG CA-3′] and reverse primer OI2c [5′-GCC TCG CGA CTT CGC AAC CCA T-3′]) [2], [9]. PCR using these primer sets from Las-infected citrus was expected to amplify specific fragments of approximately 0.5 kbp for Las606/LSS and 1.2 kbp for OI1/OI2c, respectively.

### Real-time PCR

A SYBR Premix ExTaq kit (Takara) was used to label and amplify templates for real-time PCR. Real-time PCR analyses were performed using a Stratagene Mx3000p system (Stratagene, Tokyo, Japan) following manufacturer instructions. To determine PCR efficiencies for Las606/LSS and OI1/OI2c, we diluted total DNAs from arbitrarily selected Las-infected trees in serial 10-fold ranges and measured the threshold cycle (Ct) value at each dilution. A curve was then constructed for each primer set to determine the efficiency of amplification. Real-time PCR efficiencies (*E*) were calculated from the given slopes according to the equation *E* = 10^(−1/slope)^−1, where *E* is in the range from 0 (minimum value; i.e., no amplification) to 1 (theoretical maximum and optimum) [21]. The extraction efficiency of template DNAs with each sample preparation method (mean of relative values in each sample preparation method/mean of relative values in *Extracted DNA*) and detection sensitivity between the 2 primer sets (mean of relative values in Las606/LSS primer set/mean of relative values in OI1/OI2c primer set) were calculated according to delta-Ct methods [21].

### FISH assays and oligonucleotide probes

The following oligonucleotides were used for FISH assays: (1) Las specific, an almost identical oligonucleotide to PCR primer LSS [18], (LSS; 5′-CCC AAC ATC TAG GTA AAA ACC TAA ACT TGA-3′); (2) α-proteobacteria-specific (ALF968; 5′-GGT AAG GTT CTG CGC GTT-3′) [22]; (3) eubacteria-specific (EUB338; 5′-GCT GCC TCC CGT AGG AGT-3′) [23]; and (4) negative (NON; 5′-AGT GAC GCC GTC GA-3′) [24]. These oligonucleotides, except for NON, were designed to hybridize to part of the corresponding 16S ribosomal RNA (rRNA) region specifically. Oligonucleotides used in this study were labeled in the 3′-ends with digoxigenin (DIG)-dideoxyuridine-triphosphate with a DIG Oligonucleotide 3′-End Labeling Kit, 2nd generation (Roche Applied Science, Penzberg, Germany).

FISH assays using DIG-labeled oligonucleotides were performed as previously described [25], [26] with minor modifications. Briefly, the resuspension of precipitated pellet obtained using the Biomasher III (*Biomasher-pellet*) was further filtered with a syringe filter of 5.0 µm pore size (Whatman Puradisc 30, GE Healthcare, Tokyo, Japan). For this experiment, Las-infected citrus Ishigaki1-A and uninfected citrus were used. Each filtered sample was prepared to a volume of 400 µL with distilled water. The resulting filtered samples were fixed by adding 3 volumes (300 µL) paraformaldehyde solution (4% paraformaldehyde in phosphate-buffered saline [PBS; 130 mM sodium chloride, 10 mM sodium phosphate; pH 7.2]). After an overnight incubation, a pellet was formed via centrifugation for 2 min at 5000×*g* and resuspended in PBS. The fixed suspension was then spotted onto a glass slide and incubated to air dry for 2 h. The slide was dehydrated using a series of 50, 80, and 100% ethanol.

Fifty nanograms of labeled oligonucleotide probe in 8 µL hybridization solution (900 mM sodium chloride, 20 mM Tris-HCl, and 0.01% sodium dodecyl sulfate; pH 7.2) was applied to the fixed solution on the glass slide, and hybridization was carried out in an isotonically equilibrated humid chamber for 2 h at 45°C. After hybridization, 10 µL diluted anti-DIG-fluorescein labeled antibody (Roche) in blocking solution (150 mM sodium chloride, 100 mM Tris-HCl, and 1% blocking reagent [Roche]; pH 7.5) was applied to the slide and incubated for 1 h at 27°C in the humid chamber. After incubation with washing solution (150 mM sodium chloride, 100 mM Tris-HCl, and 0.01% sodium dodecyl sulfate; pH 7.4) for 10 min at 29°C, 5 µL 4′,6-diamidino-2-phenylindole (DAPI) solution (10 µg/µL) was added to the slide for 10 min at room temperature. The slide was rinsed adequately with distilled water, and the fixed suspension was observed using Axioplan2 epifluorescent microscopy (Carl Zeiss, Jena, Germany) with a Plan-APOCHROMAT 100×/1.4 Oil objective (Carl Zeiss).

## Results

### Direct PCR using templates obtained using various preparation methods

**Figure 1:**
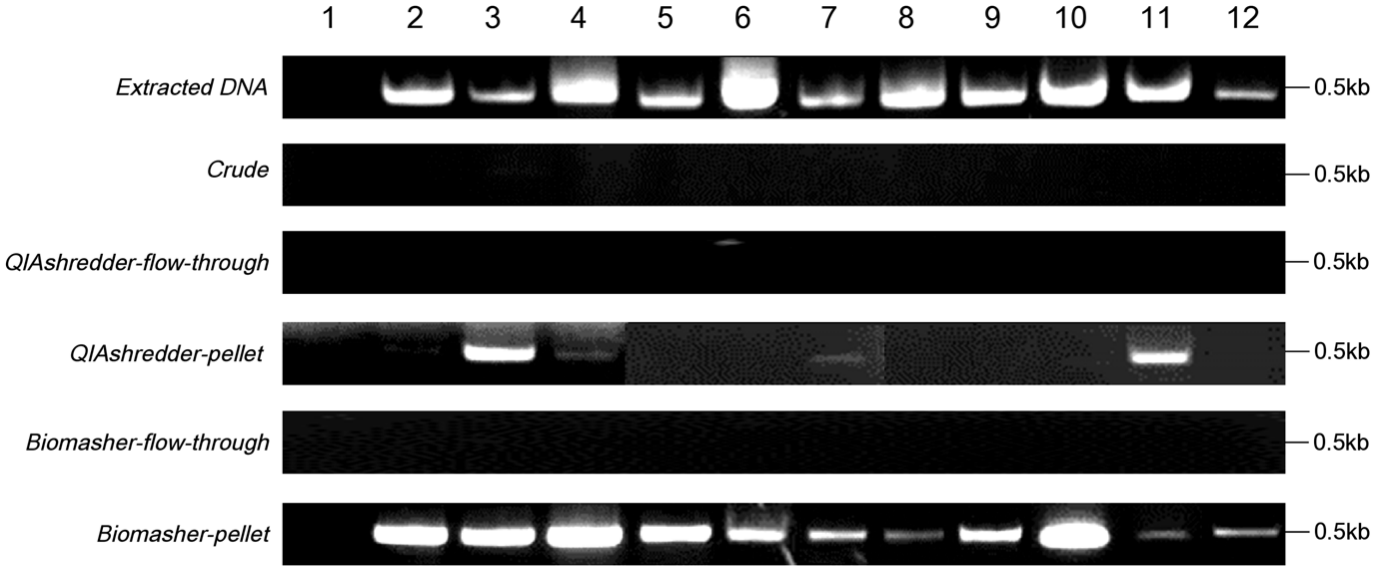
Assays of direct and conventional PCR. Templates obtained using the six preparation methods were used for direct and conventional PCR. Amplicons of each PCR were confirmed using 1.5% agarose gel electrophoresis. Lane 1, Healthy; lane 2, Ishigaki1-A; lane 3, Ishigaki1-B; lane 4, Ishigaki1-C; lane 5, Kin1-A; lane 6, Kin1-B; lane 7, OK901-A; lane 8, OK901-B; lane 9, Ishigaki3-A; lane 10, Ishigaki3-B; lane 11, Ishigaki4-A; and lane 12, Miyako13-A.

To the achievement of PCR detection of Las without DNA extraction, it is necessary that Las bacterial cells are effectively extracted and concentrated from infected leaves. Hence, we investigated the feasibility of direct PCR with templates obtained using various preparation methods. The samples from 11 Las-infected leaves and one healthy citrus leaf (see Fig. S1) were prepared as PCR templates using various methods. Direct PCR was attempted with all of these samples except the *Extracted DNA* sample, whereas conventional PCR was performed on the *Extracted DNA* sample only. The primer set Las606/LSS was chosen because conventional PCR using this primer set has higher sensitivity and more robustness than those of other commonly used primer sets, including OI1/OI2c [18]. In leaf samples from 11 Las-infected citrus, conventional PCR with *Extracted DNA* was consistently successful (see Fig. 1), concurrently, direct PCR with *Biomasher-pellet* templates from 11 Las-infected citrus was also successful (see Fig. 1). Direct PCR with *QIAshredder-pellet* templates was successful in 4 of 11 Las-infected citrus (i.e., Ishigaki1-B, Ishigaki1-C, OK901-A, and Ishigaki4-A), which are symptomatic visibly (see Fig. 1). However, direct PCR failed with all templates designated *Crude*, *QIAshredder-flow-through*, and *Biomasher-flow-through* (see Fig. 1). PCR with healthy citrus samples never resulted in amplification, as expected (see Fig. 1). In addition, PCR without templates or *ExTaq* polymerase never resulted in amplification (data not shown). From the results of direct PCR, the pellet solutions obtained using Biomasher III and QIAshredder were considered feasible templates for direct PCR. In particular, the precipitated pellet samples obtained using Biomasher III (*Biomasher-pellet*) were the most robust templates for direct PCR.

### Comparison of extraction efficiency among the preparation methods and detection sensitivity of primer sets

We found that the precipitated pellet samples obtained with the Biomasher III and QIAshredder were feasible templates for direct PCR. However, the extraction efficiency of the template DNAs contained in the pellets and detection sensitivity of PCR using each primer set seemed to differ in relation to the preparation method. To clarify this data, we compared the extraction efficiency of three methods (*Extracted DNA*, *Biomasher-pellet*, and *QIAshredder-pellet*) and detection sensitivity of two primer sets (Las606/LSS and OI1/OI2c) by calculating the relative Ct values derived from real-time PCR assay. After real-time PCR using two primer sets with templates obtained using three methods, each Ct value was derived using software in a Stratagene Mx3000p system. To avoid the misinterpretation of Ct value owing to nonspecific amplification or primer-dimer formation, we designated 30 cycles as the upper limit of Ct. Incidentally, the Ct value of sample from uninfected citrus was not determined by 40 cycles (data not shown).

**Figure 2:**
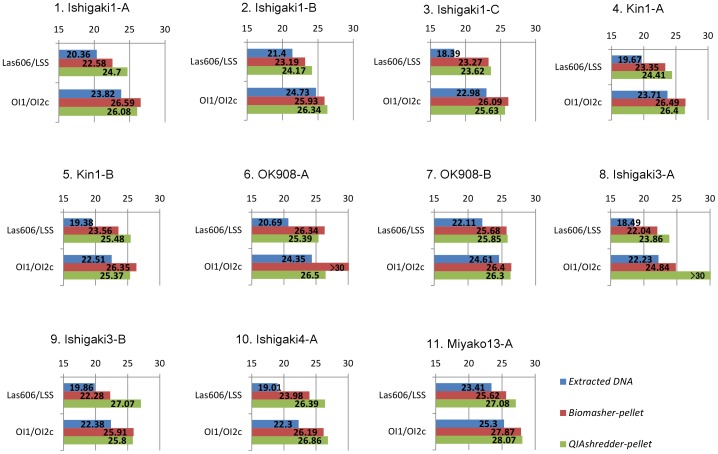
View of threshold (Ct) values derived from real-time PCR. Ct values were compared among PCR amplifications using three preparation methods and two primer sets. In each of 11 Las-infected citrus leaf samples, real-time PCR using Las606/LSS and OI1/OI2c primer sets with templates obtained from *Extracted DNA*, *Biomasher-pellet*, and *QIAshredder-pellet* was performed to derive each Ct value. The numbers in each graph are Ct values.

The Ct values of each sample from all 11 Las-infected citrus were determined (Fig. 2). The *E* of each primer set was calculated as described [21], and both were approximately 1.0 (1.00±0.01 [Las606/LSS] and 0.99±0.02 [OI1/OI2c]), regardless of preparation method. Therefore, the extraction efficiency of the three methods and detection sensitivity of the two primer sets were calculated relatively using the delta-Ct method [21]. The Ct values were processed to relative values based on PCR samples using the OI1/OI2c primer set with the *Extracted DNA* template (see Table S1). The extraction efficiencies of *Biomasher-pellet* and *QIAshredder-pellet* relative to those of the *Extracted DNA*, which were calculated by combining both primer sets, were approximately 0.1 (0.14±0.10 [*Biomasher-Pt*] and 0.12±0.10 [*QIAshredder-Pt*], respectively]. Moreover, the detection sensitivity of Las606/LSS relative to that of OI1/OI2c in each method sample was approximately 6.5 (10.07±5.54 [*Extracted DNA*], 6.84±4.11 [*Biomasher-pellet*], and 2.13±1.37 [*QIAshredder-pellet*]).

Based on this efficiency and sensitivity, PCR using Las606/LSS with all samples of *Extracted DNA* was considered to have higher power of detection (mean is 11.02 fold and median is 10.06 fold, relatively; see Table S1). Also, five samples of *Biomasher-pellet* and one sample of *QIAshredder-pellet* showed higher values than the benchmark value of 1.00, which is the PCR value using OI1/OI2c with *Extracted DNA* (see Table S1). PCR using Las606/LSS with *Biomasher-pellet* samples showed similar values as that of PCR using OI1/OI2c with *Extracted DNA* samples (see Table S1). Meanwhile, PCR using Las606/LSS with *QIAshredder-pellet* samples, PCR using OI1/OI2 with *Biomasher-pellet* samples, and PCR using OI1/OI2c with *QIAshredder-pellet* samples had values lower than that of PCR using OI1/OI2c with samples of *Extracted DNA* (approximately 0.46 fold [*QIAshredder-pellet*, Las606/LSS], 0.15 fold [*Biomasher-pellet*, OI1/OI2c], and 0.16 fold [*QIAshredder-pellet*, OI1/OI2c]). From these results, we concluded that although the power of detection was highest in conventional PCR using Las606/LSS with *Extracted DNA* as the template, direct PCR using Las606/LSS with *Biomasher-pellet* samples provided adequate power of detection for practical use.

### Detection of Las bacterium using FISH assay

**Figure 3:**
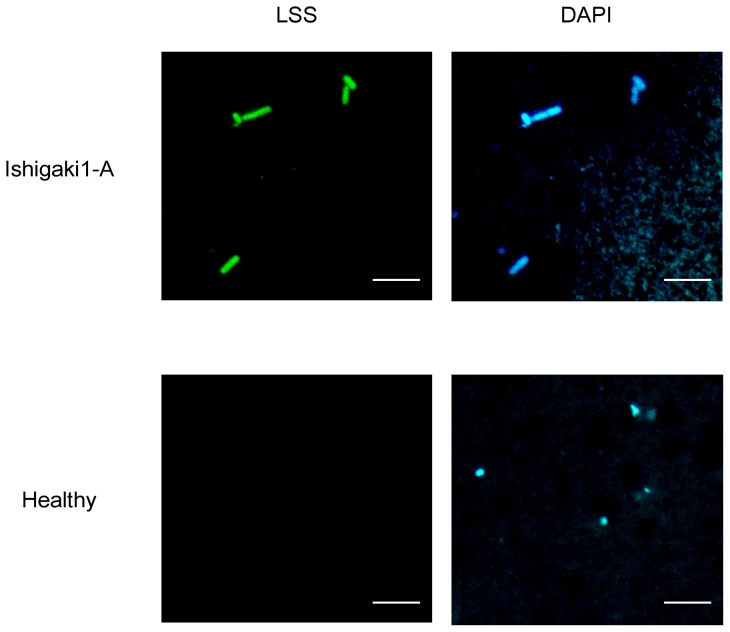
Fluorescence *in situ* hybridization (FISH) assay with Las-specific probe. The *Biomasher-pellet* from Ishigaki1 Las-infected leaves or healthy leaves was filtered with 5-µm and used for FISH assay to detect Las bacterium bodies. Upper panels indicate Ishigaki1-A Las-infected leaves, and lower panels indicate healthy leaves. Left panels, FISH with Las-specific probe, LSS. Right panels, 4′,6-diamidino-2-phenylindole (DAPI) staining. Scale bar indicates 5 µm.

The results of above direct PCR suggested that the resuspensions of precipitated pellet samples obtained using the mini homogenizer tube Biomasher III (*Biomasher-pellet*) contained Las bacterial cells. To examine the existence of Las bacterial cells in the *Biomasher-pellet*, the samples from Ishigaki1-A Las-infected citrus and healthy citrus were first used for FISH assay. Because these *Biomasher-pellet* samples included impurities such as leaf debris, adequate clarified images of the assay were not acquired. Thus, these *Biomasher-pellet* samples were filtered with a 5-µm syringe filter to remove impurities. The 5-µm filtered samples were used for FISH assay and examined closely under a microscope. LSS for Las 16S rRNAs, an oligonucleotide that is almost identical to PCR primer LSS (see Materials and Methods), ALF968 for α-proteobacteria 16S rRNAs [22], EUB338 for eubacteria 16S rRNAs [23], and NON, which is an invalid probe [24], were used as FISH probes. After FISH assay with an LSS probe, rod-shaped and atypical rod-shaped spots were detected with both the fluorescein-conjugated anti-probe antibody and DAPI (Fig. 3; Fig. S2). Similar spots were also detected with these fluorescein-conjugated anti-probe antibodies in the FISH assay using probes targeting α-proteobacteria and eubacteria (see Fig. S2). Because the Las bacterium belongs to the α-proteobacteria family of eubacteria, these rod-shape spots were considered Las bacterial cells. The estimated size of Las single cells ranged from approximately 2 to 4 µm in length. Some cells, which had various shapes, were detected with FISH assay using probes targeting α-proteobacteria and eubacteria both in the Ishigaki1-A sample and the healthy leaf sample (see Fig. S2; Fig. S3). Because these bacteria were not detected by the LSS probe, it is thought to be indigenous bacteria and endophytes in citrus.

### Effect of fixation of samples before preparation of *Biomasher-pellet* on direct PCR

**Figure 4:**
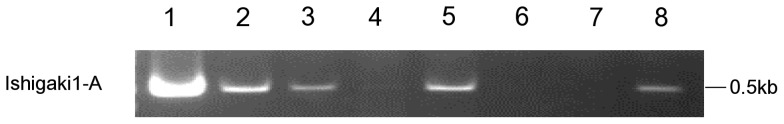
Assay of direct PCR with *Biomasher-pellet* preparation after fixation. The midribs of Ishigaki1-A Las-infected leaves were divided into equal weight aliquots and fixed with respective fixation chemicals. After fixation, templates for direct PCR were prepared using the *Biomasher-pellet* method. Amplicons of each PCR were confirmed with 1.5% agarose gel electrophoresis. Templates were as follows: lane 1, *Extracted DNA* (for conventional PCR); lane 2, *Biomasher-pellet* without fixation; lane 3, *Extracted DNA* after *Biomasher-pellet* (for conventional PCR); lane 4, *Biomasher-pellet* after −20°C freezing; lane 5, *Biomasher-pellet* after 70% ethanol fixation; lane 6, *Biomasher-pellet* after FAA (formalin 1∶acetic acid 1∶70% ethanol 8) fixation; lane 7, *Biomasher-pellet* after 4% formalin fixation; and lane 8, *Biomasher-pellet* after RNA*later* fixation.

In practical quarantine, many samples from suspected trees are collected for diagnosis. In some cases, collected samples may need to be fixed to prevent secondary infection from these samples at the inspection location. This practice is mandatory, for example, when leaves are sent from greening-infested areas to greening-free areas in Japan. Therefore, we examined whether the fixation of cells affected the preparation of *Biomasher-pellet*. The following chemicals were selected to fix the bacterial cells: freezing at −20°C, 70% ethanol, FAA (formalin 1∶acetic acid 1∶70% ethanol 8), 4% formalin in PBS, and RNA*later* (Ambion, Austin, TX). Approximately 0.05 g midribs of Ishigaki1-A was fixed using each chemical for 1 day, and subsequently each template was prepared using the Biomasher III for direct PCR. Also, DNA samples extracted both directly from the midribs and freshly from a *Biomasher-pellet* sample were used as templates for conventional PCR for comparison. As expected, conventional PCR with *Extracted DNA*, as well as direct PCR with *Biomasher-pellet* and *Extracted DNA* after *Biomasher-pellet* were successful (Lane 1, 2, and 3 in Fig. 4). Furthermore, direct PCR with *Biomasher-pellet* after fixation in 70% ethanol and with *Biomasher-pellet* after fixation in RNA*later* was also successful (Lane 5 and 8 in Fig. 4). Thus, 70% ethanol and RNA*later* were useful in fixation of samples for *Biomasher-pellet*.

## Discussion

Although citrus greening disease is subject to surveillance worldwide to prevent the expansion of the infested areas, few cases of successful containment or eradication have been reported. The main reasons for this failure may be that infected trees overlooked by inspectors become reservoirs for a second infection. To prevent this problem, a large and comprehensive survey is indispensable. Therefore, the incorporation of time- and cost-saving procedures when a large number of samples are tested is crucial.

In this study, we found that direct PCR, which greatly reduces time and cost, was applicable for the detection of Las DNA. Although the preparation of the templates for direct PCR using mini homogenizing tubes or shredder spin columns (Biomasher III and QIAshredder in this study, respectively) is necessary, the costs of these techniques are much lower than those of various kits for DNA extraction (in our estimation of various catalog prices). Moreover, the preparation time for direct PCR templates is much shorter than that required to extract DNA for conventional PCR, allowing inspectors to handle more suspected trees within an allotted time. The performance of the direct PCR using *Biomasher-pellet* reported herein is not inferior to that of conventional PCR. In fact, the efficiency of detection with direct PCR using *Biomasher-pellet* with the Las606/LSS primer set exceeds that of conventional PCR with the commonly used primer set OI1/OI2c.

Both the initial concentration of template DNA and the sensitivity of primers influence the total efficiency of any PCR systems. In this study, the initial concentration of template DNA was estimated from Ct values of real-time PCR under the supposition that the difference in Ct values of real-time PCR within the same primer set correlates to the initial concentration of template DNA. In the case of using either the Las606/LSS or the OI1/OI2c primer set, both the estimated initial concentration of template DNA of *Biomasher-pellet* and *QIAshredder-pellet*, which can be used for direct PCR, were approximately 10-fold lower than that obtained through the *Extracted DNA* method. Meanwhile, in the same sample, the difference in Ct value depends on the sensitivity of the primer set annealing to target DNAs in the PCR reaction. The sensitivity of PCR with the *Extracted DNA* template using the Las606/LSS primer set was approximately 10-fold higher than that obtained using the OI1/OI2c primer set. Therefore, conventional PCR with Las606/LSS was useful for diagnosis with high accuracy as described in a previous study [18]. Furthermore, because the sensitivity of direct PCR using Las606/LSS with *Biomasher-pellet* was almost the same as that of conventional PCR using OI1/OI2c, direct PCR using Las606/LSS with *Biomasher-pellet* is adequate for diagnosis. In contrast, although the extraction efficiencies of *Biomasher-pellet* and *QIAshredder-pellet* by real-time PCR analysis had almost no difference, the amplicons of direct PCR using *QIAshredder-pellet* with the Las606/LSS primer set in infected but asymptomatic samples could not be confirmed in electrophoresis. This reason is still unclear. However, when the preparation using QIAshredder spin column will be modified, the sensitivity of detection may be improved.

The preparation of templates for direct PCR using mini homogenizer tubes such as Biomasher III is very easy. Complete grinding of midribs with a mortar and pestle, which is required for a typical DNA extraction, is unnecessary. Interestingly, even when the midribs were briefly ground and left in visible pieces, the pellet obtained with the Biomasher III (*Biomasher-pellet*) could be used as the template for direct PCR. This outcome may be explained by the release of many viable Las bacterial cells from the sieve elements of the Biomasher III, which is supported by the results of the FISH assay.

In this study, the filtration of *Biomasher-pellet* using the syringe filters of 5 µm pore sizes was used for the FISH assay. As expected, the Las-specific FISH probe captured rod-shape bacterial cells in the samples from Las-infected leaves. Intriguingly, Las cells were observed individually but never in clusters, suggesting that Las might exist at low concentrations in sieve elements of infected trees, as has been estimated with quantitative PCR and observed with microscopy in previous studies [16], [17], [27]. Because the Las bacterium belongs to the α-proteobacteria family of eubacteria, Las bacterial cells were also detected using FISH probes specific to α-proteobacteria and eubacteria. Some organisms detected in healthy leaves by these eubacteria- and α-proteobacteria-specific probes were apparently indigenous bacteria and bacterial endophytes. The new method reported herein, consisting of viable Las bacteria extraction from the fresh leaf midribs using Biomasher III and direct PCR, is highly applicable for the diagnosis of greening. In the field, samples collected from suspected trees are often fixed to preserve Las DNAs and avoid secondary infection. In this study, five fixation methods were tried, and two methods (i.e., 70% ethanol fixation and RNA*later*) did not affect results on direct PCR. In conclusion, a combination of techniques described here (70% ethanol or RNA*later* fixation of samples, Las bacteria extraction with Biomasher III, and direct PCR using the Las606/LSS primer set) results in rapid detection of greening suitable for large-scale diagnosis.

## Supporting Information

Table S1 **Relative values of detection efficiency.**
(DOCX)

Figure S1 **Appearances of collected leaves.** Totally 11 Las-infected and 1 healthy citrus leaves were tested. From individual trees, 3 leaves were collected and used for preparation of templates for PCRs. Of these leaves, Ishigaki1-B, Ishigaki1-C, Kin1-B, OK908-A, and Ishigaki4-A were symptomatic. Other leaves were asymptomatic like as healthy leaves.
(PPTX)

Figure S2 **FISH assay with various probes.** The *Biomasher-pellet* from Ishigaki1-A Las-infected leaves were filtered with 5 µm and used for FISH assay. Left panels, FISH with respective probes; right panels, DAPI staining. Scale bars indicate 10 µm. A, LSS (Las-specific) probe; B, ALF968 (α-proteobacteria-specific) probe; C, EUB338 (eubacteria-specific) probe; D, NON (invalid) probe. Arrows indicate the Las bacterial cells.
(PPTX)

Figure S3 **FISH assay with various probes.** The *Biomasher-pellet* from healthy leaves were filtered with 5 µm and used for FISH assay. Left panels, FISH with respective probes; right panels, DAPI staining. Scale bars indicate 10 µm. A, LSS (Las-specific) probe; B, ALF968 (α-proteobacteria-specific) probe; C, EUB338 (eubacteria-specific) probe; D, NON (invalid) probe.
(PPTX)
