# Supplementary material for: Convenient Detection of the Citrus Greening (Huanglongbing) Bacterium ‘Candidatus Liberibacter asiaticus’ by Direct PCR from the Midrib Extract
Source: PLoS One. 2013 Feb 20;8(2):e57011. doi: 10.1371/journal.pone.0057011 (PMC3577761; doi:10.1371/journal.pone.0057011)
Supplement: Table S1 — Relative values of detection efficiency. (DOCX) [file pone.0057011.s001.docx]

# **Table S1. Relative values of detection efficiency**

|  | Ishigaki1-A | | Ishigaki1-B | | Ishigaki1-C | | Kin1-B | | Kin1-C | | OK901-A | | OK901-B | | Ishigaki3-A | | Ishigaki3-B | | Ishigak4-A | | Miyako13-A | | Mean | | Median | |
| --- | --- | --- | --- | --- | --- | --- | --- | --- | --- | --- | --- | --- | --- | --- | --- | --- | --- | --- | --- | --- | --- | --- | --- | --- | --- | --- |
|  | Las606 /LSS | OI1/ OI2c | Las606 /LSS | OI1/ OI2c | Las606 /LSS | OI1/ OI2c | Las606 /LSS | OI1/ OI2c | Las606 /LSS | OI1/ OI2c | Las606 /LSS | OI1/ OI2c | Las606 /LSS | OI1/ OI2c | Las606 /LSS | OI1/ OI2c | Las606 /LSS | OI1/ OI2c | Las606 /LSS | OI1/ OI2c | Las606 /LSS | OI1/ OI2c | Las606 /LSS | OI1/ OI2c | Las606 /LSS | OI1/ OI2c |
| *Extracted  DNA* | 11.00 | 1.00 | 10.06 | 1.00 | 24.08 | 1.00 | 16.45 | 1.00 | 8.75 | 1.00 | 12.64 | 1.00 | 5.66 | 1.00 | 13.36 | 1.00 | 5.74 | 1.00 | 9.78 | 1.00 | 3.71 | 1.00 | 11.02 | 1.00 | 10.06 | 1.00 |
| *Biomasher- Pellet* | 2.36 | 0.15 | 2.91 | 0.44 | 0.82 | 0.12 | 1.28 | 0.15 | 0.48 | 0.07 | 0.25 | 0.00 | 0.48 | 0.29 | 1.14 | 0.16 | 1.07 | 0.09 | 0.31 | 0.07 | 0.80 | 0.17 | 1.08 | 0.15 | 0.82 | 0.15 |
| *QIAshredder- Pellet* | 0.54 | 0.21 | 1.47 | 0.33 | 0.64 | 0.16 | 0.62 | 0.15 | 0.13 | 0.14 | 0.49 | 0.23 | 0.42 | 0.31 | 0.32 | 0.00 | 0.04 | 0.09 | 0.06 | 0.04 | 0.29 | 0.15 | 0.46 | 0.16 | 0.42 | 0.15 |

The shading boxes indicate higher values than the benchmark value of 1.00, which is the PCR value using OI1/OI2c with *Extracted DNA.*
