# Supplementary material for: Convenient Detection of the Citrus Greening (Huanglongbing) Bacterium ‘Candidatus Liberibacter asiaticus’ by Direct PCR from the Midrib Extract
Source: PLoS One. 2013 Feb 20;8(2):e57011. doi: 10.1371/journal.pone.0057011 (PMC3577761; doi:10.1371/journal.pone.0057011)
Supplement: Figure S1 — Appearances of collected leaves. Totally 11 Las-infected and 1 healthy citrus leaves were tested. From individual trees, 3 leaves were collected and used for preparation of templates for PCRs. Of these leaves, Ishigaki1-B, Ishigaki1-C, Kin1-B, OK908-A, and Ishigaki4-A were symptomatic. Other leaves were asymptomatic like as healthy leaves. (PPTX) [file pone.0057011.s002.pptx]

## Slide 1
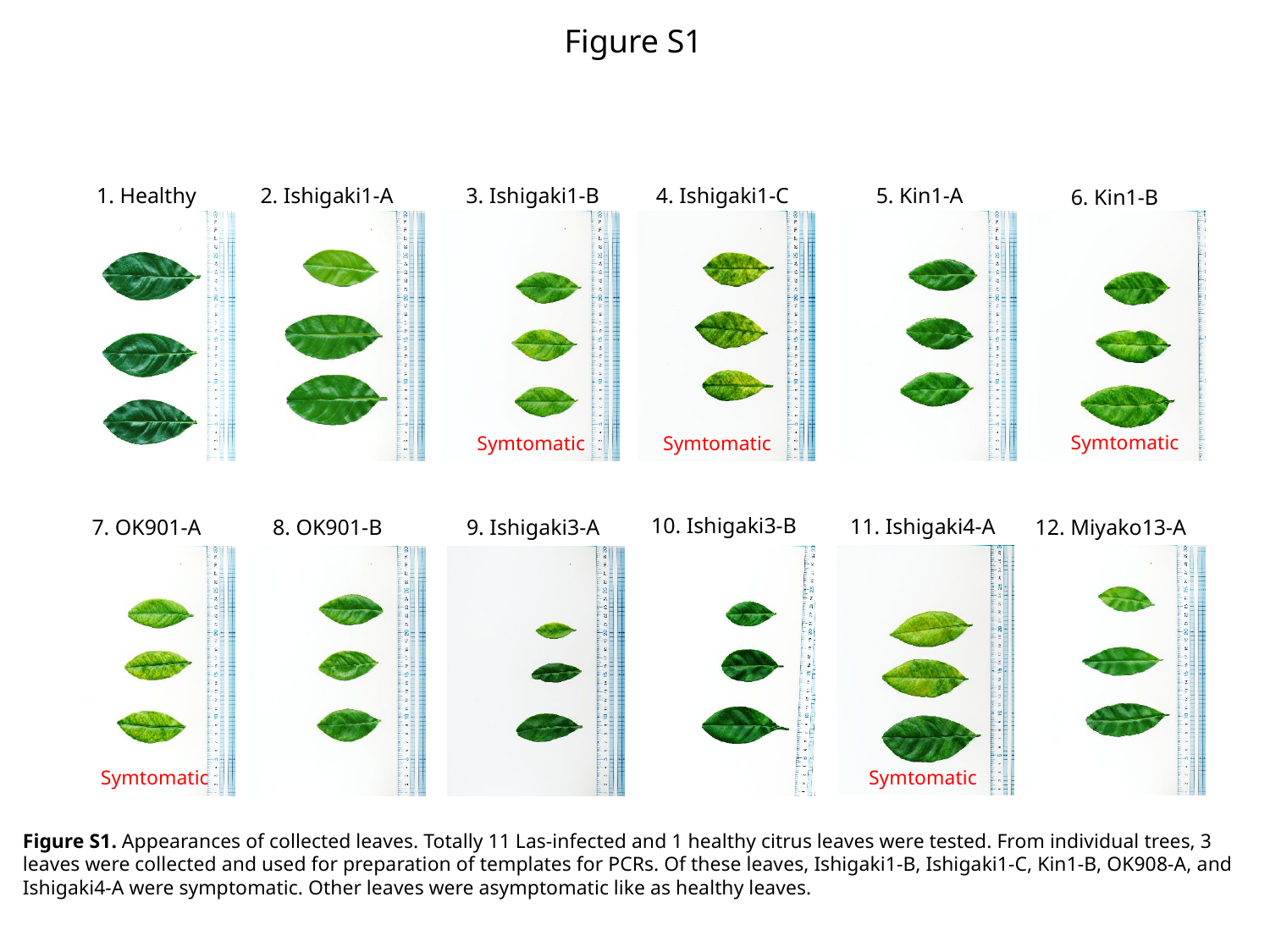

Figure S1
1. Healthy
2. Ishigaki1-A
3. Ishigaki1-B
4. Ishigaki1-C
5. Kin1-A
6. Kin1-B
Symtomatic
Symtomatic
Symtomatic
10. Ishigaki3-B
11. Ishigaki4-A
12. Miyako13-A
7. OK901-A
8. OK901-B
9. Ishigaki3-A
Symtomatic
Symtomatic
Figure S1. Appearances of collected leaves. Totally 11 Las-infected and 1 healthy citrus leaves were tested. From individual trees, 3 leaves were collected and used for preparation of templates for PCRs. Of these leaves, Ishigaki1-B, Ishigaki1-C, Kin1-B, OK908-A, and Ishigaki4-A were symptomatic. Other leaves were asymptomatic like as healthy leaves.
