# Supplementary material for: Convenient Detection of the Citrus Greening (Huanglongbing) Bacterium ‘Candidatus Liberibacter asiaticus’ by Direct PCR from the Midrib Extract
Source: PLoS One. 2013 Feb 20;8(2):e57011. doi: 10.1371/journal.pone.0057011 (PMC3577761; doi:10.1371/journal.pone.0057011)
Supplement: Figure S2 — FISH assay with various probes. The Biomasher-pellet from Ishigaki1-A Las-infected leaves were filtered with 5 µm and used for FISH assay. Left panels, FISH with respective probes; right panels, DAPI staining. Scale bars indicate 10 µm. A, LSS (Las-specific) probe; B, ALF968 (α-proteobacteria-specific) probe; C, EUB338 (eubacteria-specific) probe; D, NON (invalid) probe. Arrows indicate the Las bacterial cells. (PPTX) [file pone.0057011.s003.pptx]

## Slide 1
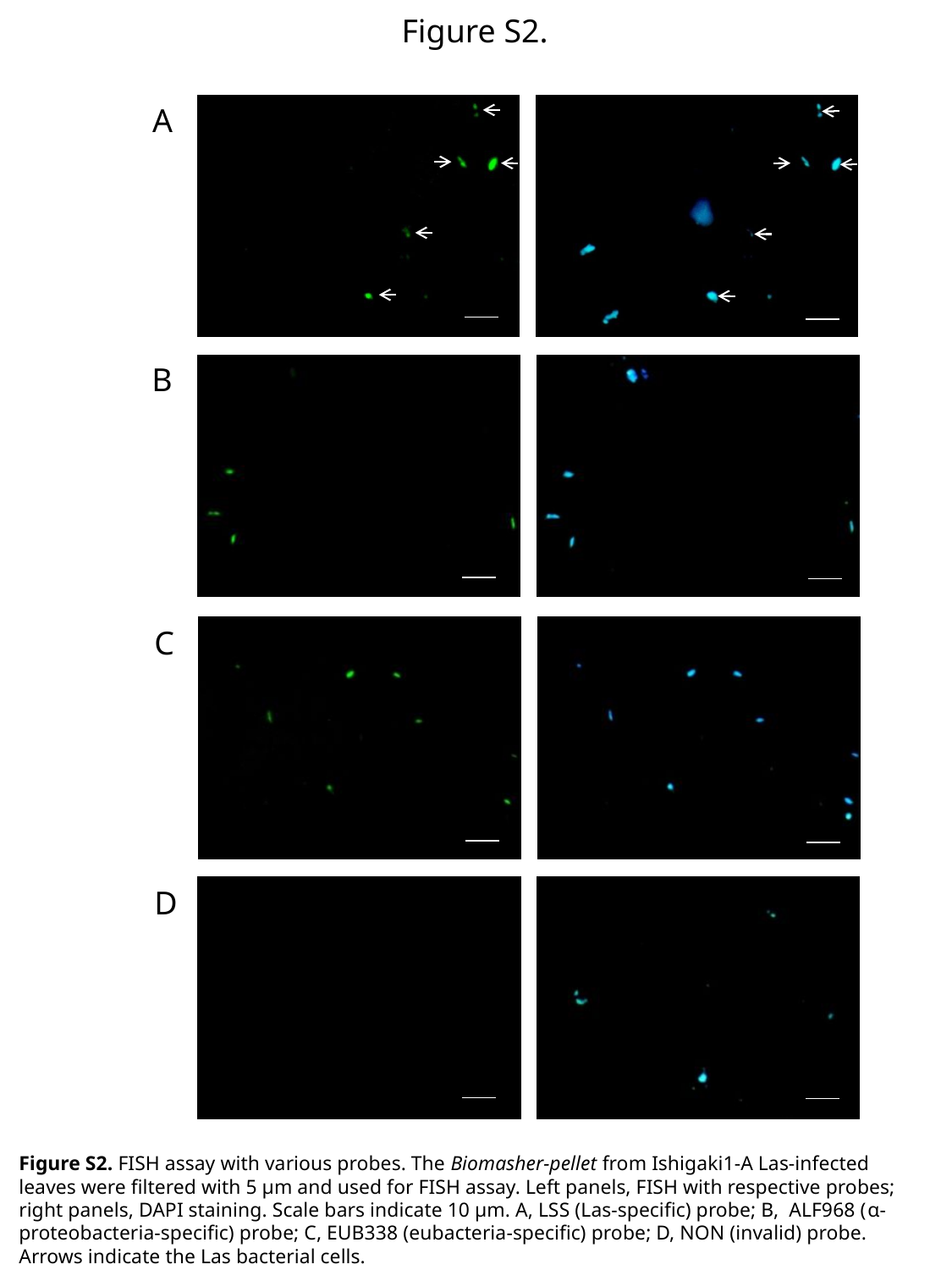

Figure S2.
A
B
C
D
Figure S2. FISH assay with various probes. The Biomasher-pellet from Ishigaki1-A Las-infected leaves were filtered with 5 µm and used for FISH assay. Left panels, FISH with respective probes; right panels, DAPI staining. Scale bars indicate 10 µm. A, LSS (Las-specific) probe; B, ALF968 (α-proteobacteria-specific) probe; C, EUB338 (eubacteria-specific) probe; D, NON (invalid) probe. Arrows indicate the Las bacterial cells.
